# Single nucleotide variants in nuclear pore complex disassembly pathway associated with poor survival in osteosarcoma

**DOI:** 10.3389/fgene.2024.1303404

**Published:** 2024-03-18

**Authors:** James E. Jacobs, Lara Davis, Shannon McWeeney

**Affiliations:** Oregon Health & Science University, Portland, OR, United States

**Keywords:** osteosarcoma, single nucleotide variants, genomic instability, nuclear pore complex, differential gene expression, MYC-overexpression

## Abstract

**Introduction:**

The bone tumor, osteosarcoma, remains challenging to treat in children and young adults, especially when patients present with metastatic disease. Developing new therapies based on genomic data from sequencing projects has proven difficult given the lack of recurrent genetic lesions across tumors. MYC overexpression has been associated with poor outcomes in osteosarcoma. However, other genomic markers of disease severity are lacking.

**Materials and Methods:**

We utilized whole genome sequencing of 106 tumors and matched normal controls in order to define genomic characteristics that correlate with overall survival. Single nucleotide variants were overlaid onto annotated molecular pathways in order to define aberrant pathway signatures specific to aggressive osteosarcoma. Additionally, we calculated differential gene expression in a subsample of 71 tumors. Differentially expressed genes were then queried for known MYC-responsive genes.

**Results:**

Molecular pathways specific to nuclear pore complex disassembly (NPCD) show significant correlation with poor overall survival in osteosarcoma when mutations were present. Genes involved in immune response and immune regulation are enriched in the differential expression analysis of samples with and without NPCD pathway aberrations. Furthermore, neither MYC nor MYC-responsive genes show differential expression between NPCD-aberrant and non-aberrant groups. The NPCD pathway mutations are dominated by regulatory region variants rather than protein-altering mutations, suggesting that dysregulation of genetic regulatory networks may be the underlying mechanism for their relation to osteosarcoma phenotype.

**Discussion:**

Overall survival is significantly worse in patients whose tumors show aberrations in the NPCD pathway. Moreover, this difference in survival is not driven by MYC-overexpression, suggesting a novel mechanism for some aggressive osteosarcomas. These findings add light to the evolving understanding of the drivers of osteosarcoma and may aid in the search for new treatments based on patient-specific genetic data.

## Introduction

The bone tumor, osteosarcoma, continues to be a significant burden to the health and wellbeing of children and young adults, with about 500 new cases per year in the United States. Overall survival (OS) at 10 years is approximately 65% for those with localized disease *versus* 25% for the one in five patients that present with metastatic disease (Voute et al., 1999; Bielack et al., 2002; Kager et al., 2003; Ferrari et al., 2005). Survival rates for osteosarcoma–utilizing multi-agent chemotherapy and surgical local control–have not improved significantly over the last 2 decades (Mirabello et al., 2009). Moreover, osteosarcoma survivors experience late effects related to therapy that can include cardiomyopathy, hearing loss, kidney dysfunction, second malignancies, infertility as well as significant physical limitations secondary to surgery (Ward et al., 2014). Thus, research leading to an increased biological understanding of this devastating tumor and development of new treatment options represents a vital need in the pediatric population and a potentially high-impact addition to our knowledge of sarcoma biology.

Studying the genomics of osteosarcoma has proven challenging, as individual tumors show tremendous heterogeneity characterized by structural variants (SVs) and copy number alterations (CNAs) (Chen et al., 2014; Perry et al., 2014). This complex genomic landscape has made it difficult to identify common mutations across patients. However, some consistent findings have emerged. Large scale sequencing studies have revealed recurrent alterations in the tumor suppressor genes, TP53 and RB1, as well as in known cancer-associated genes such as ATRX and DLG2 (Chen et al., 2014). Furthermore, common cancer-associated pathways such as the phosphatidylinositol 3-kinase/mammalian target of rapamycin (PI3K/mTOR) pathway and insulin-like growth factor (IGF) signaling have also been found to be abnormal in osteosarcoma (Perry et al., 2014; Behjati et al., 2017). While these alterations are dominated by SVs, it should be noted that germline and somatic single nucleotide variants (SNVs) have also been identified in these genes (Martin et al., 2012; Chen et al., 2014). Another consistent finding in osteosarcoma is the relationship between MYC overexpression and tumor progression leading to poor overall survival (Gamberi et al., 1998; Scionti et al., 2008; Shimizu et al., 2010; Chen et al., 2018).

Genomic instability, leading to SVs and CNAs, has been implicated as the driving force in the development of osteosarcoma (Chen et al., 2014) and specific types of genomic instability have been described in many osteosarcomas. These include the localized hypermutable regions associated with SVs, known as kataegis (Nik-Zainal et al., 2012; Chen et al., 2014), as well as massive genomic rearrangement events called chromothripsis (Behjati et al., 2017; Cortes-Ciriano et al., 2020). While these SVs and CNAs are not consistent or universal across all osteosarcomas, targeting of copy number variable genes, matched to a patient’s particular genomic profile has shown to be effective in patient-derived xenograft models (Sayles et al., 2019). Thus, SVs and CNAs appear to be the key factors in the oncogenesis of osteosarcoma. However, what leads to this underlying genomic instability and how this relates to other consistent genomic and clinical characteristics of osteosarcoma remains to be discovered. SNVs are often thought of as byproducts of the genomic instability of osteosarcoma and are believed to be passenger events (Chen et al., 2014; Bousquet et al., 2016). However, it is possible that specific patterns of mutation exist that may contribute to the severity of these tumors. Here we show that by analyzing SNVs overlayed onto annotated molecular pathways across multiple patient samples, we can identify patterns in the underlying genomics of osteosarcoma that illuminate mutations in genes that correlate with previously described genomic and clinical characteristics of osteosarcoma.

## Materials and methods

### Patient enrollment and sample collection

Whole genome sequencing (WGS) and RNA sequencing (RNA-Seq) datasets were downloaded from the St Jude Cloud (McLeod et al., 2021). All sequencing data were previously obtained as part of the Pediatric Cancer Genome Project (PCGP) and the St Jude Clinical Genomics and Genomes for Kids (G4K) Clinical Pilot Project (Rusch et al., 2018). Patient consent and sample collection were conducted as part of the PCGP and G4K Clinical Pilot Project (Rusch et al., 2018). Please refer to original study for ethics committee info. We restricted our WGS analysis to human subjects in which both the tumor and matched control sequences were available. We restricted our RNA-Seq analysis to human subjects in which a matching WGS dataset was available.

### Sequence analysis and molecular pathway annotation

WGS data were analyzed using the GATK Best Practices (Broad Institute, 2020). The downloaded files were first reverted back to an unaligned format (Broad Institute, 2019). Sequencing reads were then aligned to the most recent version of the human genome (GRCh38/hg38) which incorporates population-based contigs (Li and Durbin, 2010; Broad Institute, 2017a). The aligned reads were then separated by read group and duplicate reads were marked (Cibulskis et al., 2013; Broad Institute, 2015b). To decrease erroneous mutation calling, systemically inaccurate base quality scores were recalibrated (Broad Institute, 2015a; Broad Institute, 2017b). MuTect2 was then used to identify mutations in the tumor samples relative to the matched normal samples (Cibulskis et al., 2013; Broad Institute, 2016). The Ensemble Variant Effect Predictor (VEP) (McLaren et al., 2016) was then used to classify the called somatic mutations based on their predicted effects. The final mutation set was limited to those predicted to cause 3′ or 5’ untranslated region variants, splice site variants, transcription-factor binding site variants, protein altering variants, stop loss or gain variants, coding sequence variants, start loss variants, frameshift variants or regulatory region variants.

Following VEP filtering, mutations were mapped to curated molecular pathways using Reactome. Covering over 10,000 human proteins, 13,000 biochemical reactions and 2,400 pathways, Reactome is the most comprehensive open source, manually curated biological pathway database (Milacic et al., 2012; Fabregat et al., 2016). After selecting for pathways in which the aberrant group and the non-aberrant group were represented by at least 10 samples each, we searched for pathways that distinguished patients based on overall survival.

### Survival analysis

Of the 82 patients, 81 had available clinical data for time-to-event analyses. Of those, 79 had corresponding WGS data. Overall survival was evaluated in these 79 patients. Kaplan-Meier curves were generated with R (R.C. Team, 2020; Kassambara A and Biecek, 2021; Therneau, 2020) between samples with and without aberrant NPCD. Other cancer types were also analyzed for differences in overall survival due to aberrant NPCD. Mutation and survival data for individual cancer types were obtained from the cBioPortal for Cancer Genomics (Cerami et al., 2012; Gao et al., 2013). These data were then mapped to molecular pathways using Reactome (Milacic et al., 2012; Fabregat et al., 2016). Kaplan-Meier curves were then generated between samples with and without aberrant pathways.

### Gene differential expression analysis

Expression data (RNA-Seq) were available for a subset of 71 osteosarcoma samples. These samples were analyzed as follows: First, the downloaded RNA-Seq files were reverted back to an unaligned format (Broad Institute, 2019) and aligned to the most recent version of the human genome (GRCh38/hg38) using the STAR Aligner with the “quantMode TranscriptomeSAM GeneCounts” option (Dobin et al., 2013). Gene expression values for each sample were then estimated using RSEM (Li and Dewey, 2011). Finally differential gene expression between samples with and without mutations in NPCD was calculated in R using the DESeq2 package (Love et al., 2014). To account for multiple hypotheses testing, only genes with a false discovery rate of ≤0.05 were considered to be significantly differentially expressed.

## Results

### Mutation calling from whole genome sequencing reads

**FIGURE 1:**
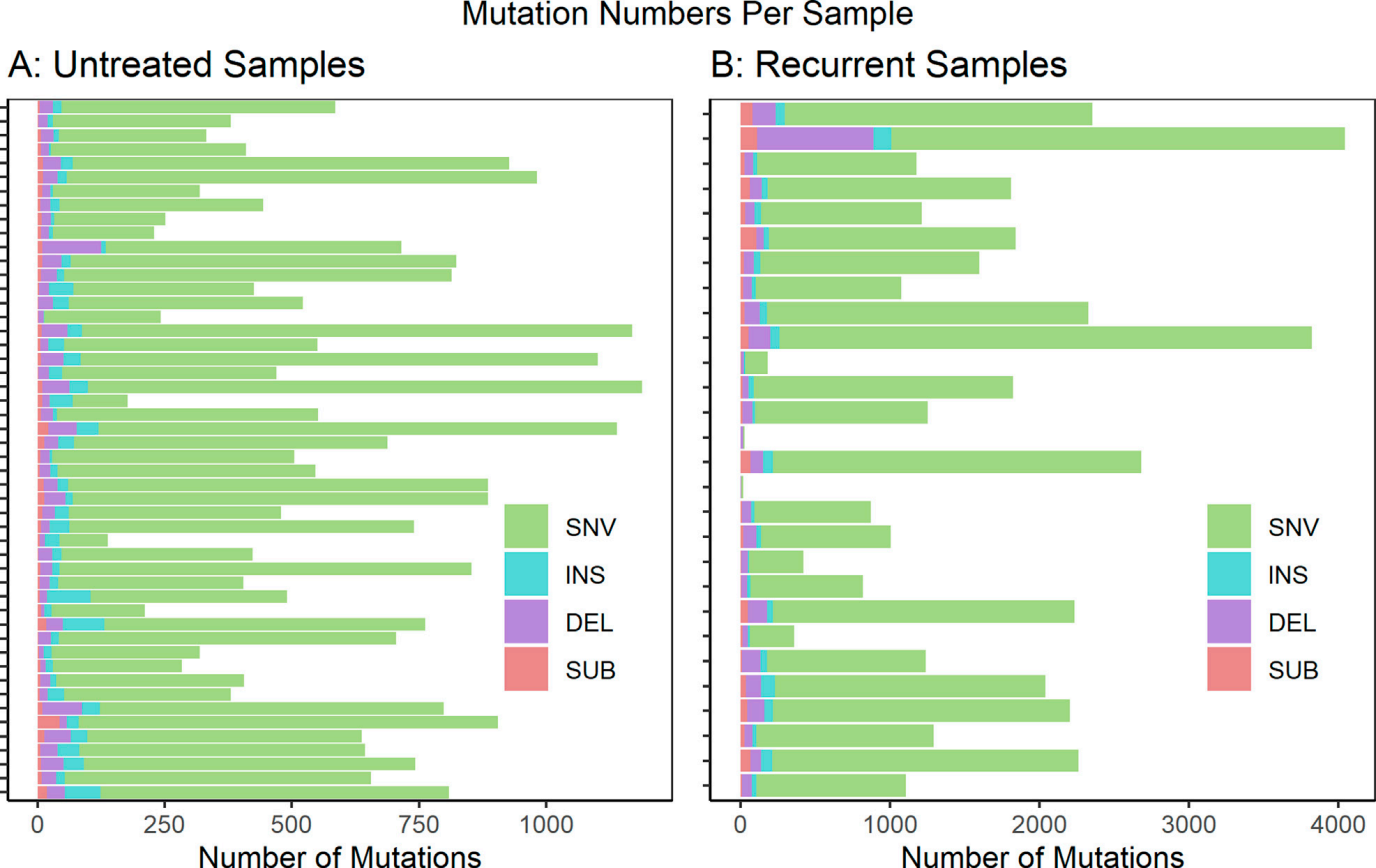
Mutations predicted to cause coding sequence (including 5′ and 3′ untranslated regions), splice site or regulatory region variants are shown per sample for **(A)** untreated samples (n = 51) and **(B)** recurrent samples (n = 28). These are further subdivided by mutation type: single nucleotide variants (SNV), insertions (INS), deletions (DEL) and substitutions (SUB). If multiple biopsy samples were taken at a unique biopsy timepoint, the mutation numbers were averaged. Not Included: autopsy biopsies (n = 7), non-recurrent treated metastatic biopsies (n = 1), treated primary biopsies (n = 19).

We analyzed WGS datasets from 81 unique patients, representing 106 total sequenced biopsies. Contamination of tumor samples was calculated at 0.0024 ± 0.00026 (Ave ± std). Somatic mutations in the tumor samples were identified relative to a matched normal sample from the same patient. The called mutations were then limited to those predicted to cause coding sequence variants (including 5′ and 3’ untranslated regions), and variants in splice sites or regulatory regions. The full list of called mutations can be found in Supplementary Material S1. The per-sample mutational burden is shown in Figure 1. The number of mutations per sample showed a wide variation with a mean and standard deviation of 605 ± 278 for untreated samples and 1,540 ± 987 for recurrent samples. The vast majority of mutations were single nucleotide variants (SNVs). However, small insertions (INS), deletions (DEL) and substitutions (SUB) were also noted. This analysis did not evaluate larger structural variants (SVs) such as copy number alterations (CNAs).

### Molecular pathway analysis

**FIGURE 2:**
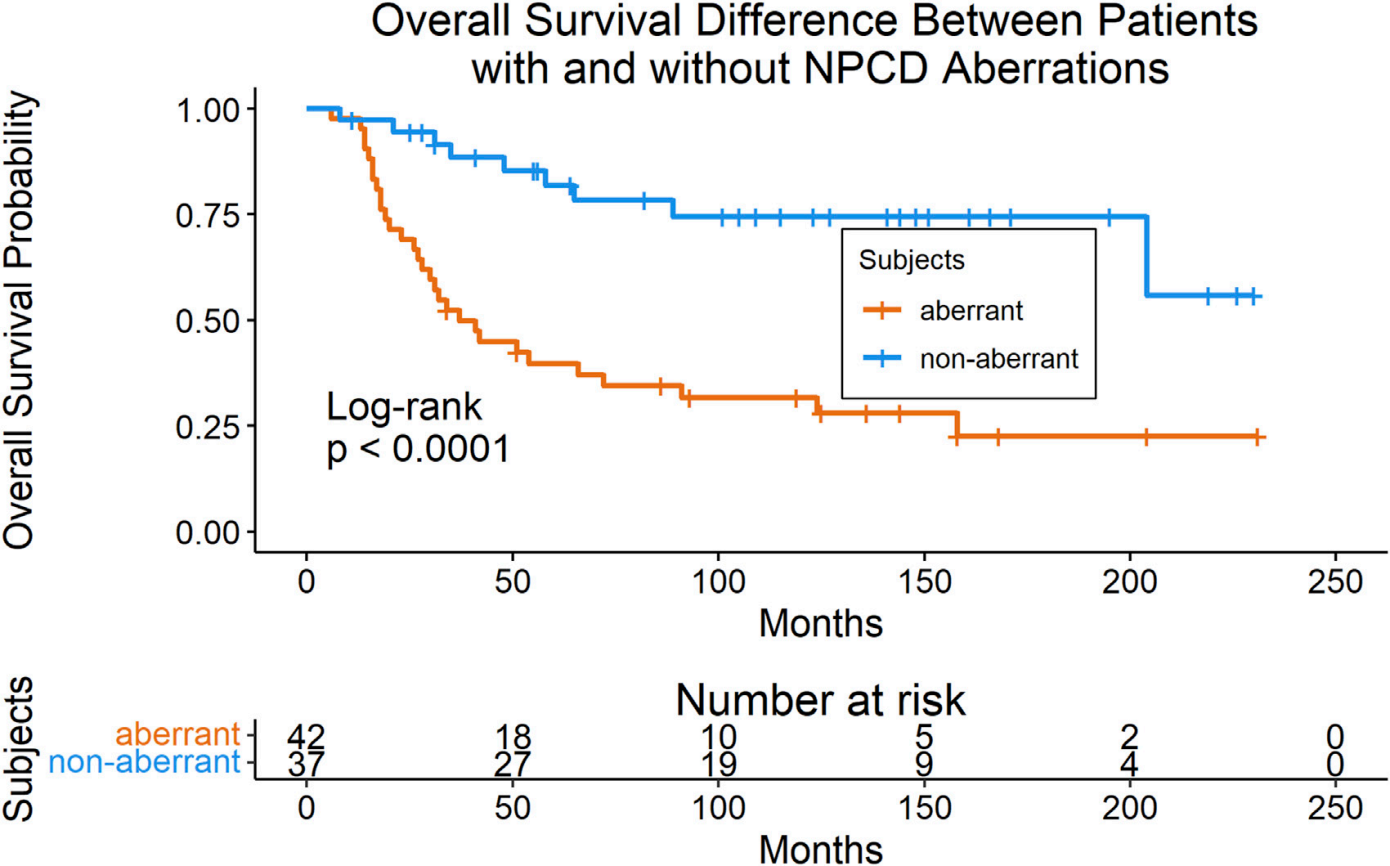
Kaplan-Meier plot (with risk table) of overall survival in patients with and without aberrant NPCD. At time point 0, there were 42 patients in the aberrant group and 37 patients in the non-aberrant group.

Mutations were mapped to annotated molecular pathways and aberrant pathways were then further analyzed in the context of clinical outcomes. Seventy-nine of the patients with mutation data also had available clinical data for time-to-event analyses (clinical data shown in Table 1). We found that patients with mutations in the nuclear pore complex disassembly (NPCD) pathway (Reactome ID: R-HSA-3301854) had significantly poorer overall survival than those without mutations (FDR = 0.03). The survival difference between groups with and without aberrant NPCD is shown in Figure 2.

**TABLE 1:** Clinical characteristics of the 79 patients with available clinical data. No statistically significant differences were detected between groups with and without NPCD aberrations with regards to patient age at diagnosis, sex, or race. A statistically significant difference was noted in duration of follow up between aberrant and non-aberrant groups as would be expected. The various histological subtypes lacked adequate numbers of samples for statistically meaningful comparison. A statistically significant difference was noted in the incidence of metastatic disease at the time of diagnosis between aberrant and non-aberrant groups and the aberrant group had more post-treatment samples available for analysis.

		Total (n = 79)	NPCD aberrant (n = 42)	NPCD non-aberrant (n = 37)	*p*-value
Age at diagnosis (years; Ave ± std)		13.6 ± 3.9	13.5 ± 4.5	13.0 ± 3.9	0.54^a^
Sex	Female	36	19	17	1**
	Male	43	23	20	
Race	Black	17	11	6	0.60***
	White	54	27	27	
	Other	8	4	4	
Tumor Histology	Conventional: NOS	16	7	9	NA
	Conventional: Osteoblastic	15	9	6	
	Conventional: Chondroblastic	4	2	2	
	Conventional: Fibroblastic	0	0	0	
	Telangiectatic	3	3	0	
	Small Cell	0	0	0	
	Mixed	3	1	2	
	Not Available	38	20	18	
Sample Type	Primary (untreated)	42	20	22	0.024***
	Post-Treatment	55	41	14	
	Metastatic (untreated)	7	4	3	
Metastatic Disease Present at Diagnosis		35	24	11^†^	0.043**
Duration of Follow Up (Months; Ave ± std)		81.0 ± 64.0	64.4 ± 58.6	103.0 ± 65.4	0.008^a^

^a^ Two-Sample *t*-test, **Chi-squared test with Yates’ continuity correction, ***Fisher’s Exact Test, ^†^Two non-aberrant subjects had unknown metastatic disease status at diagnosis, NOS: Not Otherwise Specified–Conventional osteosarcoma without subclassification into osteoblastic, chondroblastic or fibroblastic subtypes.

A full list of called mutations associated with the NPCD pathway can be found in Supplementary Material S2. No differences were noted in the variant allele frequency (VAF) between NPCD pathway mutations in untreated primary biopsy samples (0.242 ± 0.160) vs samples from recurrent lesions (0.251 ± 0.171).

As aberrations in NPCD could represent a general signature for aggressive malignancies rather than an osteosarcoma-specific signature, we analyzed overall survival in various cancer types with regards to the presence of mutations in this pathway. No differences were noted in overall survival in breast invasive carcinoma, lung squamous cell carcinoma, prostate adenocarcinoma or glioblastoma multiforme with respect to aberrations in NPCD (Supplementary Figures S1–S4) (Hoadley et al., 2018). Adult soft tissue sarcomas and Ewing sarcoma were also analyzed and again, no difference in overall survival was noted (Supplementary Figures S5, S6) (Edsc Cancer Genome Atlas Research NetworkCancer Genome Atlas Research Network, 2017). Furthermore, the sarcoma samples had very few aberrations in the NPCD pathway (Tirode et al., 2014). These findings suggest that the survival difference noted in osteosarcoma patients with aberrations in NPCD is specific to the disease process of osteosarcoma.

### Gene expression correlation

**FIGURE 3:**
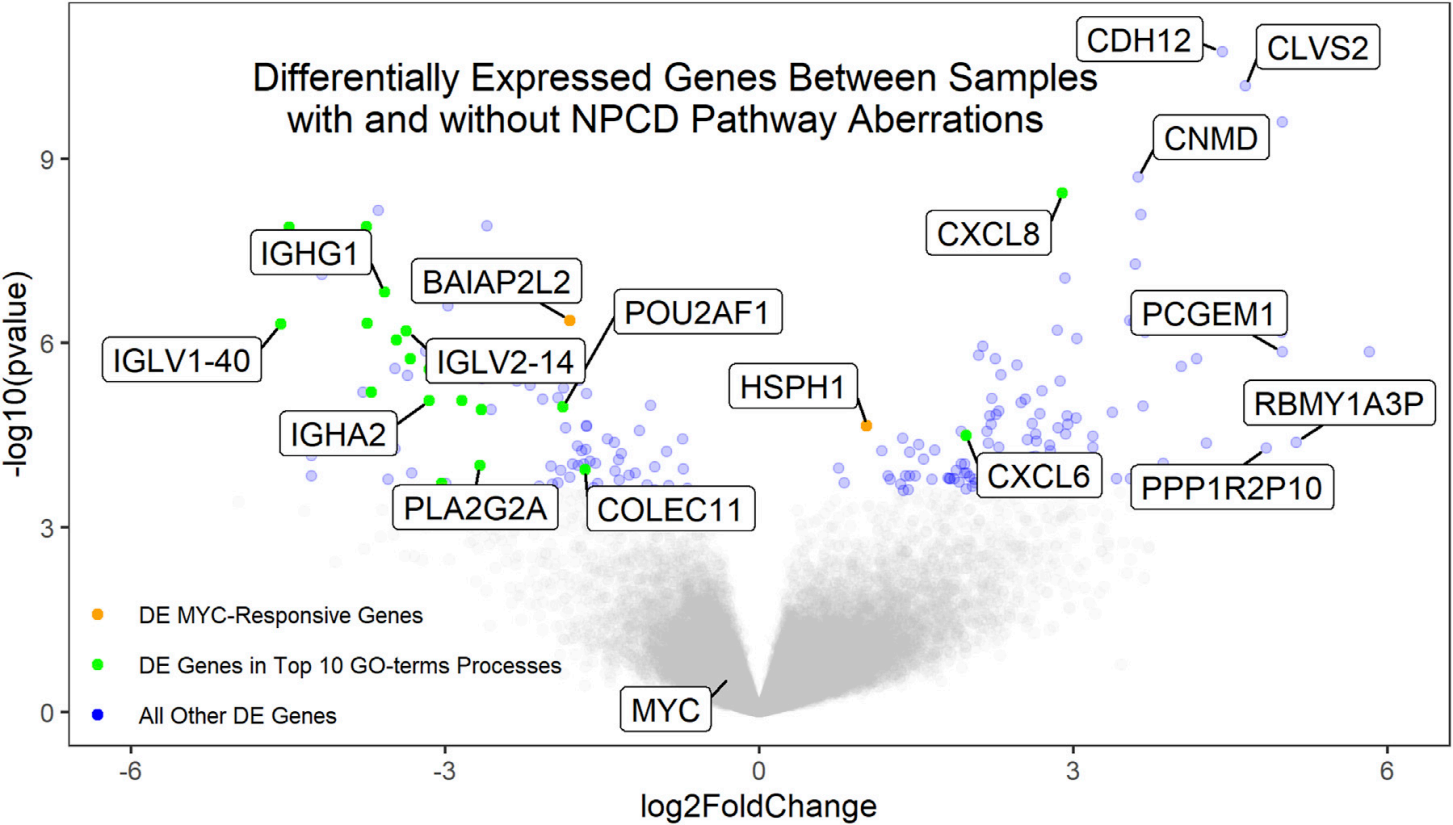
Volcano plot of the differentially expressed genes between samples with and without aberrant NPCD. Genes with positive log2FoldChange values are upregulated in samples with aberrant NPCD. Genes involved in the top 10 significantly enriched GO-terms processes (of which 8 are related to immune function) that are differentially expressed above the FDR threshold are shown in green. MYC-responsive genes differentially expressed above the FRD threshold are shown in orange. All other genes differentially expressed above the FDR threshold are shown in blue. Genes below the FDR threshold are shown in gray. Genes of interest have been labeled.

Gene expression data were available for 72 of the osteosarcoma samples. Of these, 37 showed aberrations in the NPCD pathway genes. Differential expression analysis of the aberrant vs non-aberrant groups was performed using DESeq2 (Love et al., 2014). Interestingly, neither MYC nor known MYC-responsive genes were found to be differentially expressed secondary to NPCD pathway aberrations (Figure 3). This is in contrast to MYC overexpression which was seen to be associated with poor survival (Supplementary Figure S7) as has been demonstrated in previous studies (Chen et al., 2018). These findings suggest a possible alternate mechanism for the development of aggressive osteosarcomas. Similarly, none of the NPCD pathway genes were differentially expressed between NPCD-aberrant and non-aberrant samples. A full list of differentially expressed genes can be found in Supplementary Material S3. GO-terms analysis (Eden et al., 2007; Eden et al., 2009) showed that of the 10 most significantly enriched GO-terms processes, eight relate to immune function. Notably, these genes largely showed decreased expression in the aberrant vs. the non-aberrant samples (Figure 3). A full list of GO-terms processes enriched in the differentially expressed genes can be found in Supplementary Material S4.

### Patterns in osteosarcoma nuclear pore complex mutations

**FIGURE 4:**
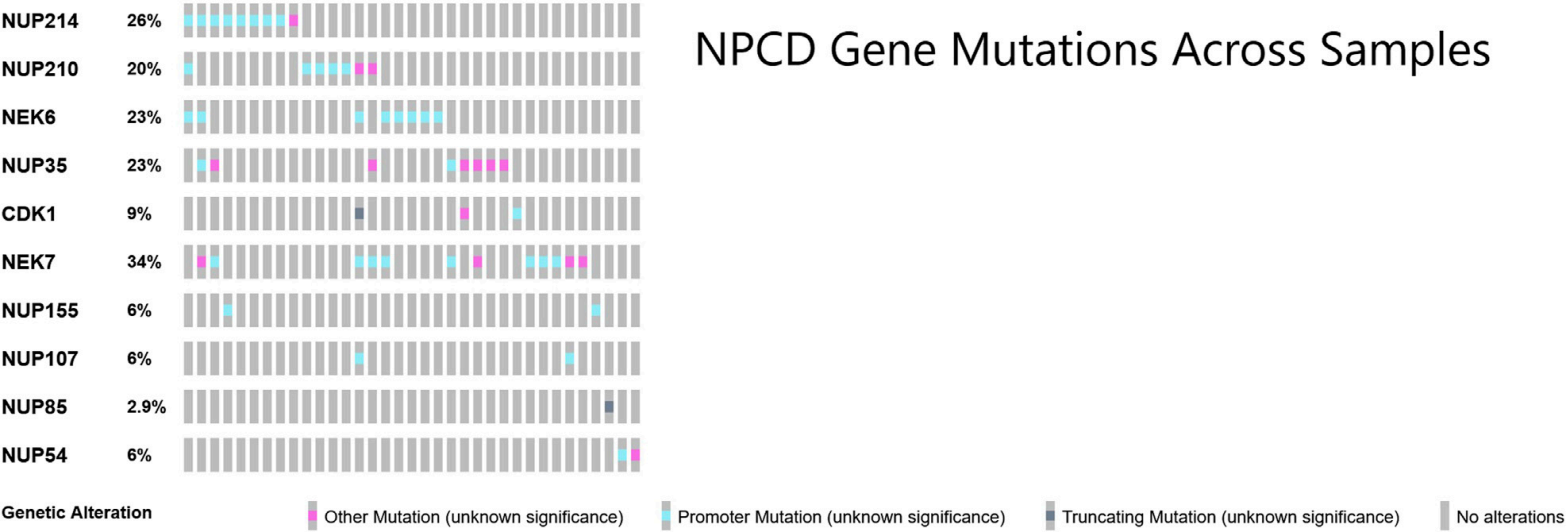
OncoPrint (Cerami et al., 2012; Gao et al., 2013) of core NPCD genes across osteosarcoma samples. Gene symbols are listed in rows and individual samples are represented as columns. There is a largely non-overlapping pattern of mutation, particularly in the promoter region mutations. Mutations included in “Other Mutation” are 3′ and 5′ untranslated region variants, transcription factor binding site variants, stop loss variants, start lost variants, frameshift variants and non-promoter regulatory region variants.

Many of the genes responsible for the regulation of nuclear pore complex disassembly were found to be mutated in this cohort of osteosarcoma samples (Laurell et al., 2011). These mutations across samples showed a largely non-overlapping pattern as shown in Figure 4. Perhaps surprisingly, the majority of the NPCD-related mutations occurred in genetic regulatory regions rather than within the coding region of the gene itself.

## Availability of data and materials

The WGS and RNA-Seq datasets analyzes for this study are available from the St Jude Cloud: https://www.stjude.cloud/

All code used in this project can be found in the following repositories:

• WGS Workflow: https://github.com/jejacobs23/osteo
• RNA-Seq Workflow: https://github.com/jejacobs23/osteo-RNA-Seq-Workflow

## Discussion

Here we show that across a cohort of osteosarcoma patients, mutations in genes responsible for the structure and function of nuclear pore complex disassembly (NPCD) portend a poorer prognosis in terms of overall survival when compared to patients without such mutations. The nuclear envelope is a crucial cellular structure in eukaryotes and is involved in a multitude of cellular processes such as the coordinated transport between the nucleus and cytoplasm, chromatin organization and regulation of gene expression (Guttinger et al., 2009). Moreover, the disassembly and reassembly of the nuclear envelope–including the nuclear pore complexes (NPCs)–is a key process in the transition between G2 and M phases of the cell cycle (Elder et al., 2000; Li et al., 2005).

Abnormalities in the NPCs have been associated with a variety of diseases including multiple types of cancer. NUP98–and to a lesser extent, NUP214–have been found to be part of various fusion events involved in the development of several different myeloid and lymphoid leukemias (Cronshaw and Matunis, 2004). Additionally, fusions involving the translocated promoter region protein (TPR) gene have been implicated in various sarcomas including osteosarcoma (Park et al., 1986; Vriens et al., 2009). Exactly how these abnormal NPC gene products are associated with malignancy is not fully understood. However, defects in the NPCs’ normal role in mitotic checkpoint regulation is an intriguing possibility (Cronshaw and Matunis, 2004). Our results highlight a downregulation of the expression of many immune-related genes in patients with NPCD aberrations. This would suggest that immune dysregulation may be a feature of more aggressive osteosarcomas, as supported by recent publications (Tang et al., 2023; Wu et al., 2023). Furthermore, the lack of protein altering mutations in the coding regions of the NPCD genes in our study suggests that dysregulation of these gene networks–rather than direct gene product dysfunction–may be responsible for the oncogenic phenotype associated with NPCD mutations in osteosarcoma.

Overexpression of the proto-oncogene, MYC, is a well-described characteristic of aggressive osteosarcomas (Chen et al., 2018). Normally, MYC is involved in the transcriptional regulation of many downstream genes. When overexpressed, this intricate cascading network becomes disrupted which can result in oncogenesis. Here we show that the differential expression of MYC and MYC-responsive genes is not present when comparing NPCD-aberrant and non-aberrant groups. These findings may point to an alternate mechanism for the development of aggressive osteosarcomas and NPCD aberrations could prove to be a valuable prognostic marker for osteosarcoma. Further studies will be required to validate these findings and determine if NPCD pathway aberrations are a suitable target for therapy.

While the difference in survival between groups of patients with and without mutations related to the NPCD genes is statistically significant, the inference of why these mutations affect prognosis is entirely speculative and cannot be resolved through purely computational analyses. Our study highlights the benefits of hypothesis-driven computational analyses guiding further bench experimentation as a way to streamline the process of discovery in the highly complex area of cancer genomics.

Acknowledgement of genetic data sources: This study makes use of data generated by the St Jude Children’s Research Hospital–Washington University Pediatric Cancer Genome Project and/or Childhood Solid Tumor Network as well as the Clinical Genomics and Genomes for Kids (G4K) Clinical Pilot.

## Data Availability

The original contributions presented in the study are included in the article/Supplementary Material, further inquiries can be directed to the corresponding author.

## Abbreviations

OS, overall survival; SVs, structural variants; CNAs, copy number alterations; SNVs, single nucleotide variants; WGS, whole genome sequencing; RNA-Seq, RNA sequencing; PCGP, Pediatric Cancer Genome Project; G4K, St Jude Clinical Genomics and Genomes for Kids; VEP, Variant Effect Predictor; NE, nuclear envelope; NPCs, nuclear pore complexes; TPR, translocated promoter region protein.
