# Supplementary figures and images for: Single nucleotide variants in nuclear pore complex disassembly pathway associated with poor survival in osteosarcoma
Source: Front Genet. 2024 Mar 18;15:1303404. doi: 10.3389/fgene.2024.1303404 (PMC10982431; doi:10.3389/fgene.2024.1303404)

R-HSA-3301854

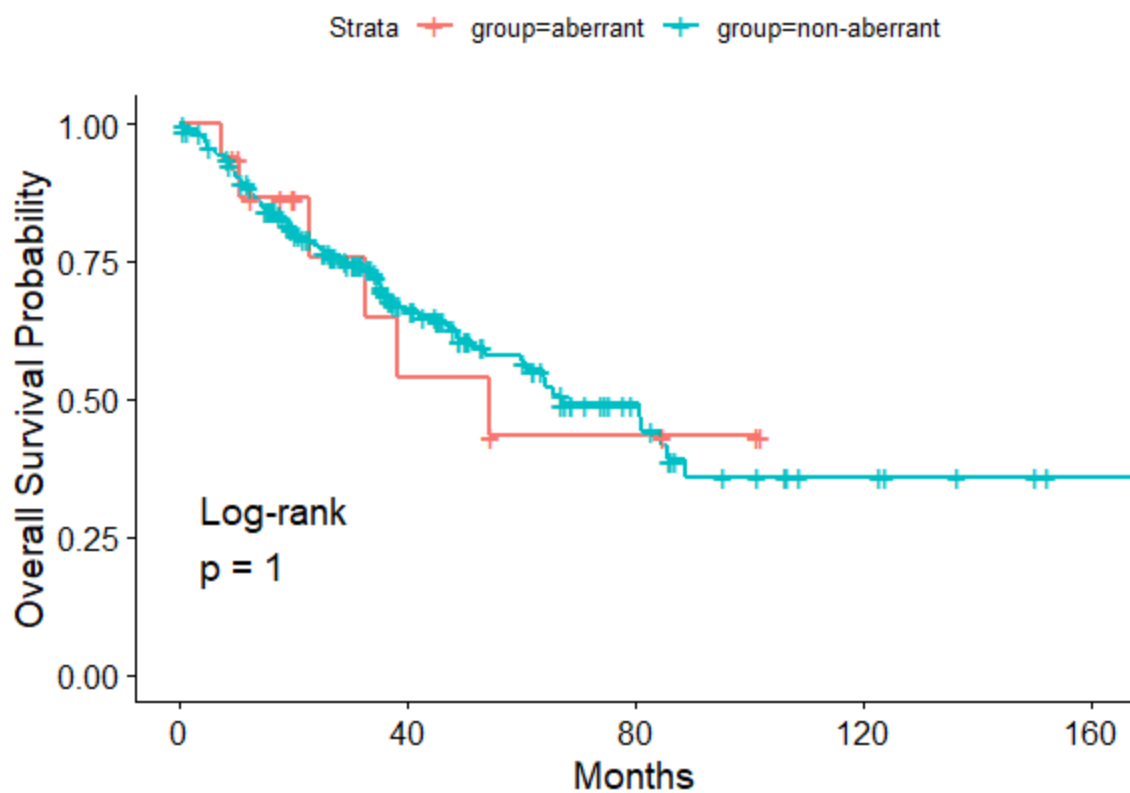

Supplement: Supplementary file 1 [file Image5.PDF]

R-HSA-3301854

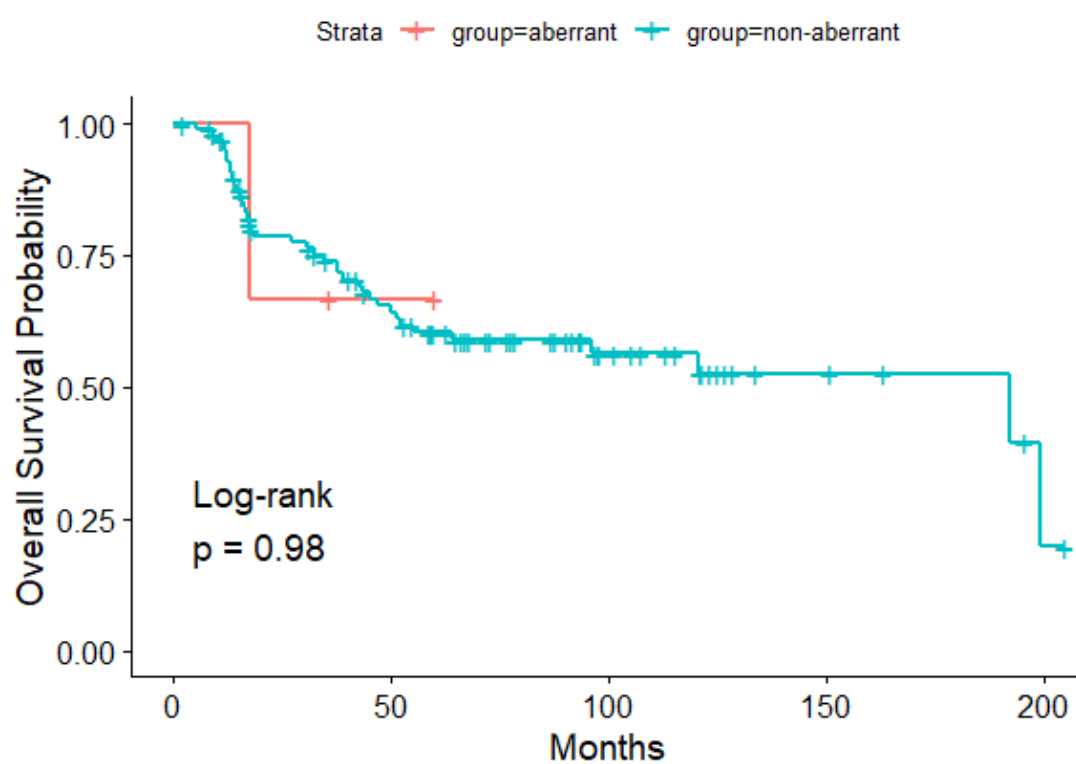

Supplement: Supplementary file 3 [file Image6.PDF]

R-HSA-3301854

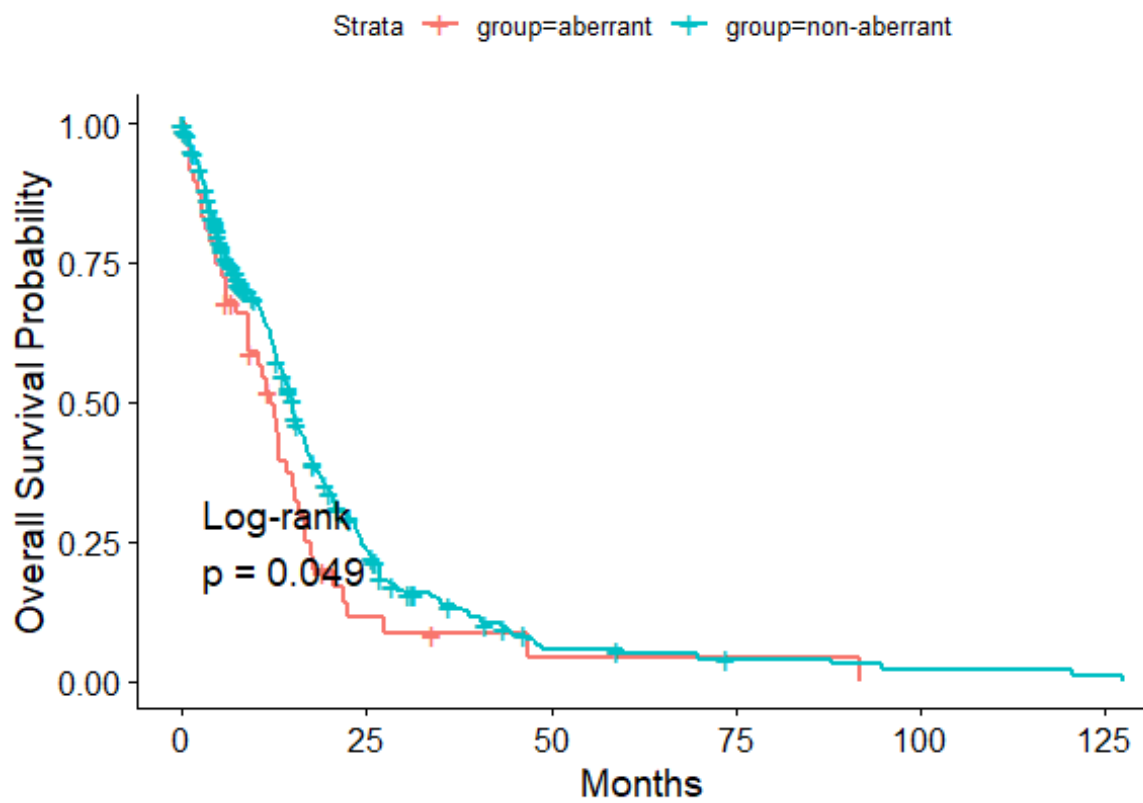

Supplement: Supplementary file 4 [file Image4.PDF]

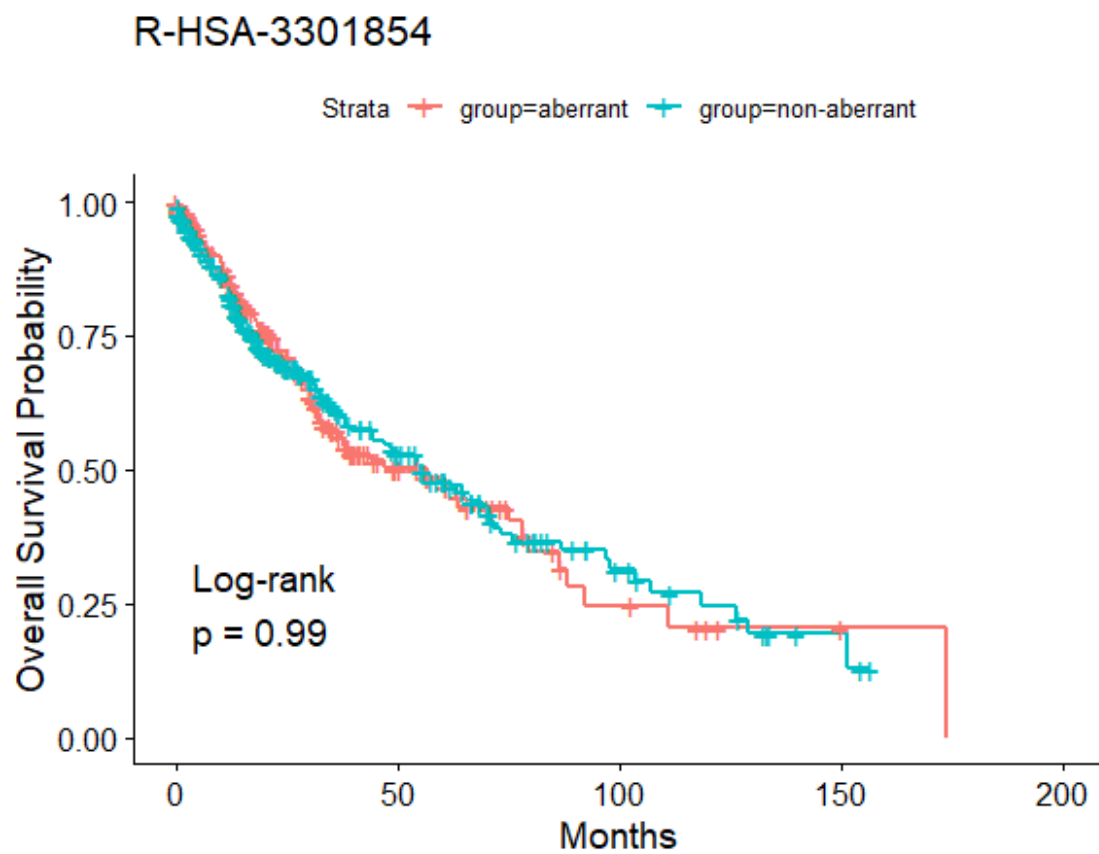

Supplement: Supplementary file 5 [file Image2.PDF]

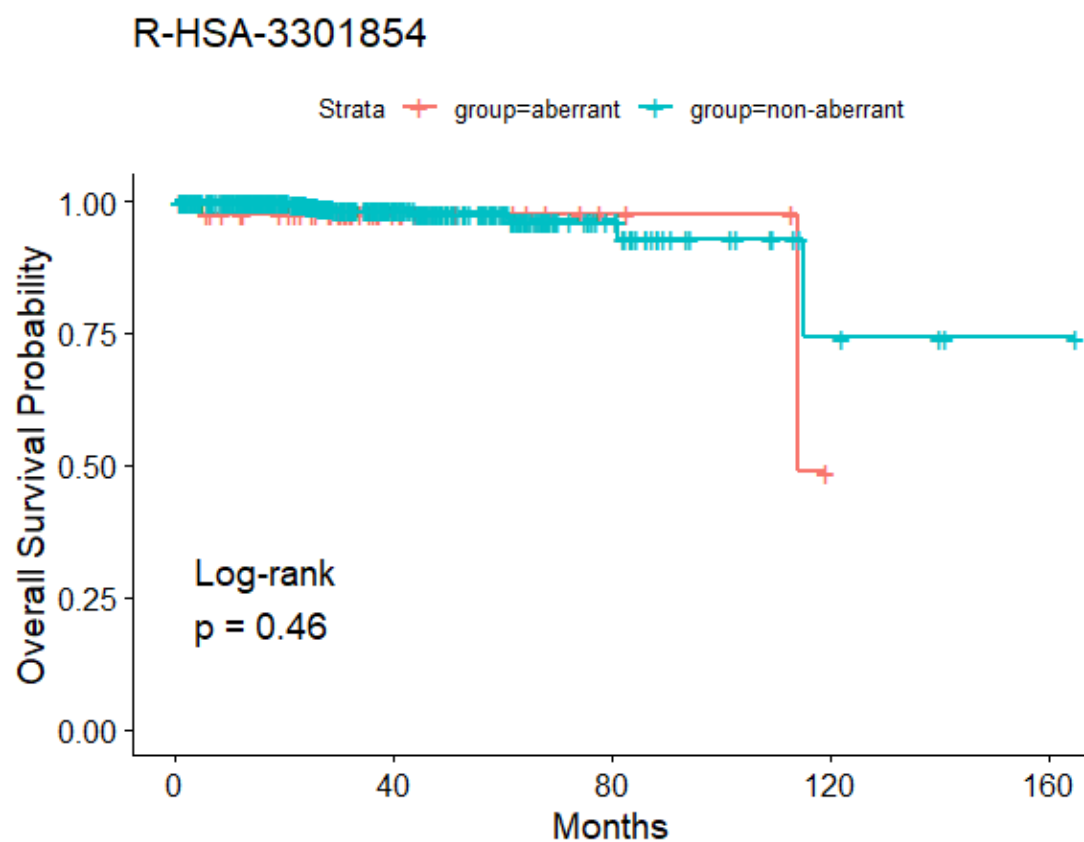

Supplement: Supplementary file 6 [file Image3.PDF]

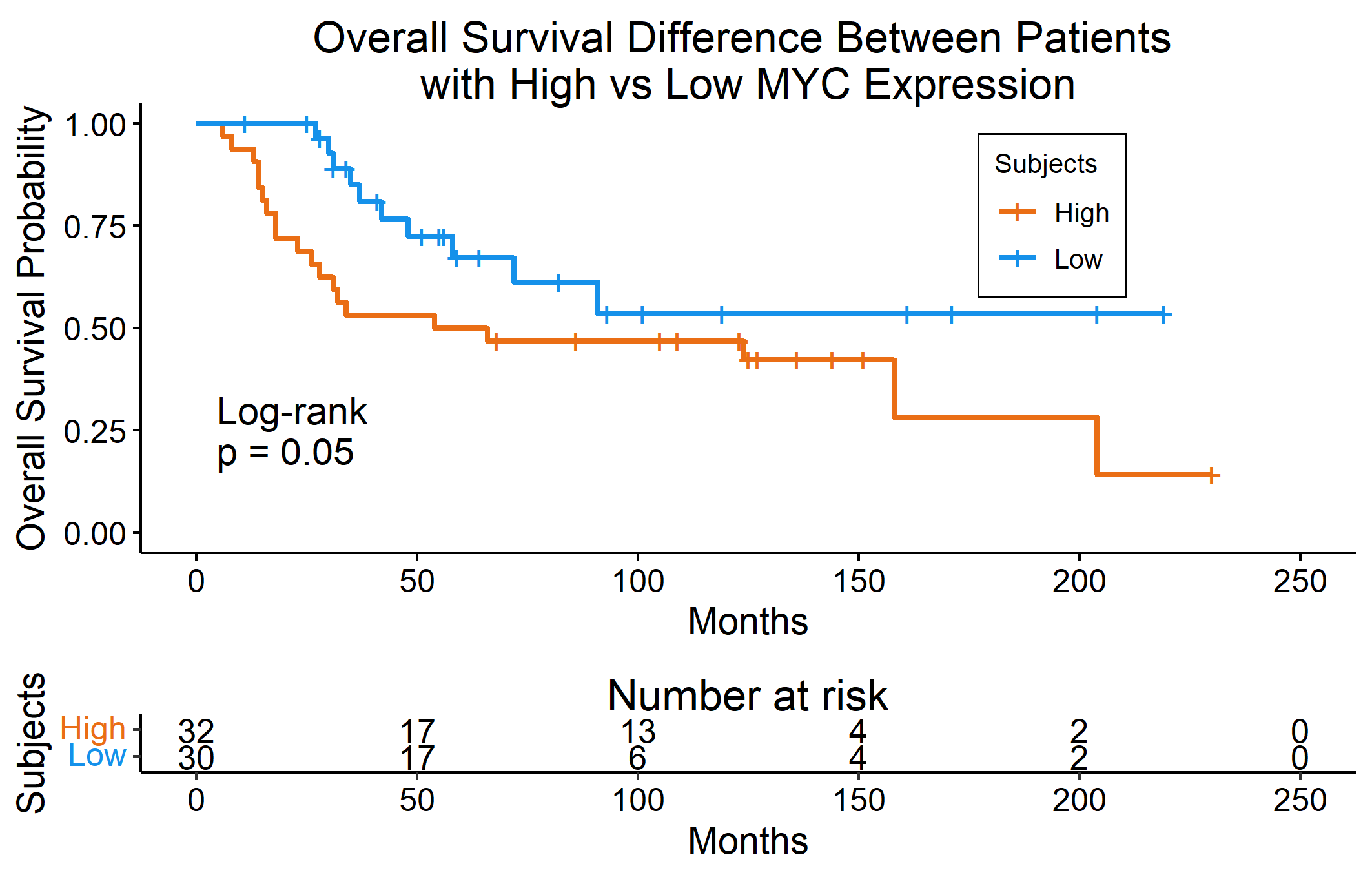

Supplement: Supplementary file 8 [file Image7.TIFF]
