# Supplementary material for: Single nucleotide variants in nuclear pore complex disassembly pathway associated with poor survival in osteosarcoma
Source: Front Genet. 2024 Mar 18;15:1303404. doi: 10.3389/fgene.2024.1303404 (PMC10982431; doi:10.3389/fgene.2024.1303404)

Overall Survival Kaplan-Meier Curves Between Samples with and without Aberrant Pathways in Breast Cancer

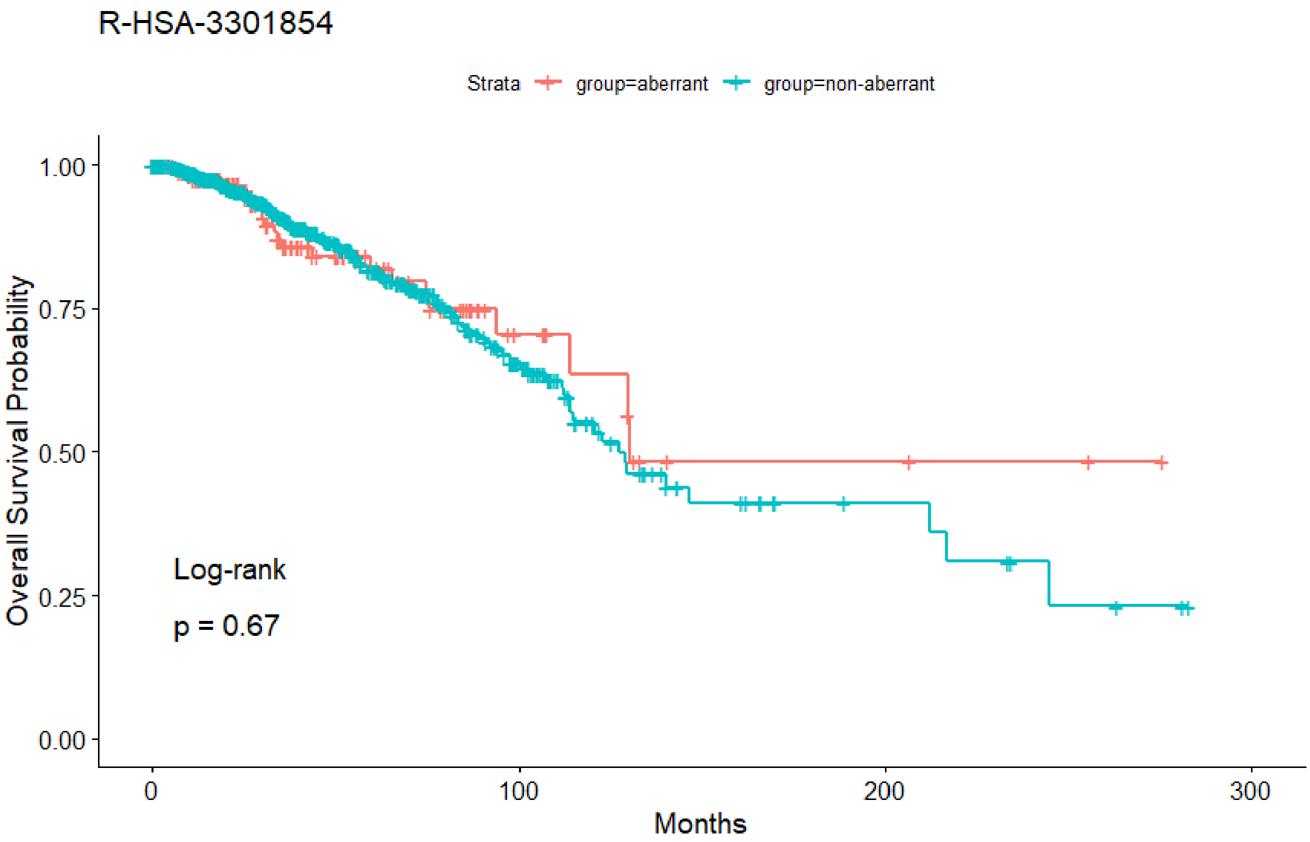

Supplement: Supplementary file 9 [file Image1.PDF]
